# Long-Term Local Injection of RAGE-Aptamer Suppresses the Growth of Malignant Melanoma in Nude Mice

**DOI:** 10.1155/2019/7387601

**Published:** 2019-09-04

**Authors:** Nobutaka Nakamura, Takanori Matsui, Yuri Nishino, Ami Sotokawauchi, Yuichiro Higashimoto, Sho-ichi Yamagishi

**Affiliations:** ^1^Department of Pathophysiology and Therapeutics of Diabetic Vascular Complications, Kurume University School of Medicine, Kurume, Japan; ^2^Department of Chemistry, Kurume University School of Medicine, Kurume, Japan; ^3^Division of Diabetes, Metabolism, and Endocrinology, Department of Medicine, Showa University School of Medicine, Tokyo, Japan

## Abstract

Accumulating evidence has suggested the pathological role of advanced glycation end products (AGEs) and their receptor RAGE axis in aging-associated disorders, including cancers. In this study, we examined the effects of local injection of RAGE-aptamer adjacent to the tumor on G361 melanoma growth in nude mice. We further investigated the effects of RAGE-aptamer on oxidative stress generation, RAGE, vascular endothelial growth factor (VEGF), and monocyte chemoattractant protein-1 (MCP-1) gene expression in *N*^*ε*^-(carboxymethyl)lysine (CML)-exposed G361 melanoma cells *in vitro*. Local injection of RAGE-aptamer adjacent to the tumor dramatically decreased the growth of G361 melanoma in nude mice, which was associated with reduced expression of CML, RAGE, nitrotyrosine, VEGF, CD31, and von Willebrand factor, markers of endothelial cells in G361 tumors. Furthermore, RAGE-aptamer inhibited the binding of CML to V-domain of RAGE and blocked the CML-induced increases in oxidative stress generation, RAGE, VEGF, and MCP-1 mRNA levels in G361 melanoma cells. Our present findings suggest that long-term local injection of RAGE-aptamer adjacent to the tumor could inhibit melanoma growth in nude mice partly by suppressing tumor angiogenesis via blockade of the CML-RAGE interaction. Local injection of RAGE-aptamer may be a feasible therapeutic tool for the treatment of malignant melanoma.

## 1. Introduction

Cancer is one of the aging-associated diseases, and its prevalence is increased among older people, especially in those with a long-term history of diabetes [1–3]. Indeed, cancer incidence has increased rapidly after age of 50 years [2]. Moreover, mortality risk of cancers is significantly higher in diabetic patients compared with nondiabetic individuals, whereas the association of diabetes with cancer death is attenuated by adjusting for glycemic parameters, such as glycated hemoglobin (HbA1c) [3].

Advanced glycation end products (AGEs) are senescent macromolecular derivatives formed progressively both at a normal aging process and under hyperglycemic conditions, which could be a marker of cumulative diabetic exposure in patients with diabetes [4]. Accumulating evidence has suggested that AGEs play a crucial role in the development and progression of various aging-associated disorders, such as cancer growth and metastasis, cardiovascular disease, osteoporosis, and neurodegenerative disorders [5–15]. Furthermore, engagement of a receptor for AGEs (RAGE) with AGEs has been shown to stimulate oxidative stress generation and evoke inflammatory and thrombotic reactions, thereby being involved in the devastating disorders [5–15]. These observations suggest that development of novel therapeutic strategy that can inhibit the AGE-RAGE axis may make a valuable contribution to increasing healthy life expectancy in our growing and aging society.

We have previously shown that DNA-aptamer raised against RAGE (RAGE-aptamer) inhibits the binding of AGEs to RAGE *in vitro* and attenuates the development and progression of diabetic nephropathy in streptozotocin-induced type 1 diabetic rats by blocking the harmful effects of AGEs in the kidneys [16]. In addition, continuous intraperitoneal infusion of RAGE-aptamer for 4 weeks has also been found to suppress the growth and liver metastasis of malignant melanoma in nude mice [17]. Since *N*^*ε*^-(carboxymethyl)lysine (CML), one of the well-characterized AGEs, is a representative ligand for RAGE [16, 18], this study investigated whether long-term local injection of RAGE-aptamer adjacent to the tumor suppressed the growth of malignant melanoma in nude mice by inhibiting the tumor-promoting effects of CML and resultantly increased the survival rate of melanoma-bearing nude mice.

## 2. Materials and Methods

### 2.1. Materials

Dulbecco's modified Eagle's medium (DMEM) and G361 melanoma cells were obtained from Sigma-Aldrich (St. Louis, MO, USA) and American Type Culture Collection (Manassas, VA, USA), respectively. CML was from Cayman Chemical (Ann Arbor, MI, USA) or Iris (Waldershof, Germany). Monoclonal antibodies (Abs) raised against CML and nitrotyrosine were purchased from TransGenic Inc. (Fukuoka, Japan) and StressMarq Biosciences Inc. (Victoria, BC, Canada), respectively. Abs raised against RAGE (H-300), vascular endothelial growth factor (VEGF) (C-1), CD31 (M-20), and von Willebrand factor (vWF) (F8/86) were obtained from Santa Cruz Biotechnology, Dallas, TX, USA. M.O.M. immunodetection kit and Histofine Simple Stain MAX-PO(MULTI) were purchased from Vector Lab., Inc. (Burlingame, CA, USA) and Nichirei Co. (Tokyo, Japan), respectively. 5-(and-6)-carboxy-2′,7′-dihydrofluorescein diacetate (carboxy-H_2_DFFDA) was purchased from Thermo Fisher Scientific (Sab Jose, CA, USA).

### 2.2. Preparation of RAGE-Aptamer

RAGE-aptamer was selected using systemic evolution of ligands by exponential enrichment (SELEX) as described previously [16]. Sequence of phosphorothioate-modified RAGE-aptamer is 5′-ccTgATATggTgTcAccgccgccTTAgTATTggTgTcTAc-3′; phosphorothioate nucleotides are indicated as capital letters. Molecular weight and melting temperature of RAGE-aptamer are 12,549 and 69.8°C, respectively.

### 2.3. G361 Malignant Melanoma Cells

G361 melanoma cells were maintained in DMEM containing 10% fetal calf serum as described previously [17]. In experiments for reactive oxygen species (ROS) generation and reverse transcription-polymerase chain reaction (RT-PCR) analysis, G361 cells were incubated with the indicated concentrations of CML in the presence or absence of the indicated concentrations of RAGE-aptamer for the indicated time periods. *In vitro*-G361 cell experiments were performed in DMEM supplemented with 1% fetal calf serum.

### 2.4. Animal Experiments

Two million G361 cells were intradermally administered into the upper flank of female athymic nude mice at 6 weeks(Japan Clea, Tokyo) (*n* = 13) as described previously [17]. Two weeks after tumor inoculation, RAGE-aptamer (38.4 pmol/day/g body weight, *n* = 6) or vehicle (*n* = 7) was injected adjacent to the tumor every day for 7 days. After 3 days off, RAGE-aptamer or vehicle was readministered adjacent to the tumor 3 days in a row. This was repeated for 15 cycles. Tumor size and body weight were determined twice a week, and tumor volume (mm^3^) was calculated by multiplying (largest diameter of tumor)/2 by (smallest diameter of tumor)^2^. At 113 days after tumor inoculation, mice were humanely sacrificed by isoflurane inhalation, and G361 melanoma was excised for immunohistochemical staining. All the animal experiments were approved by the ethnical committee of Kurume University School of Medicine, Japan, and performed according to the guide for Care and Use of Laboratory Animals of the National Institutes of Health.

### 2.5. Immunohistochemical Staining

G361 melanoma specimens were fixed, embedded, and incubated overnight at 4°C with Abs raised against CML (1 : 500 dilution), RAGE (1 : 200 dilution), nitrotyrosine (1 : 1,000 dilution), VEGF (1 : 100 dilution), CD31 (1 : 200 dilution), and vWF (1 : 200 dilution). Then, CML, nitrotyrosine, VEGF, and vWF expressions were visualized with M.O.M. immunodetection kit, while RAGE and CD31 with MAX-PO(MULTI) kit. Immunoreactivity was measured by ImageJ.

### 2.6. Quartz Crystal Microbalance (QCM) Binding Assay

The binding affinities of CML and RAGE-aptamer to human V-domain RAGE (vRAGE; residues 23–121) were measured using a sensitive 27-MHz QCM (Initium Inc., Japan) as described previously [16].

### 2.7. ROS Measurement

G361 cells were treated with or without 10 *μ*M carboxy-H_2_DFFDA for 30∼60 minutes. Then, G361 cells were incubated with the indicated concentrations of CML in the presence or absence of the indicated concentrations of RAGE-aptamer for 25 minutes. Intracellular ROS production in G361 cells was measured with a fluorescent probe, carboxy-H_2_DFFDA as described previously [17].

### 2.8. RT-PCR

Quantitative real-time RT-PCR was performed using Assay-on-Demand and TaqMan 5 fluorogenic nuclease chemistry (Thermo Fisher Scientific) according to the supplier's recommendation. IDs of primers for human RAGE, VEGF, monocyte chemoattractant protein-1 (MCP-1), *β*-actin, and 18S rRNA genes were Hs00542584_g1/Hs00542592_g1, Hs03929005_m1/Hs00900055_m1, Hs00234140_m1, Hs01060665_g1, and Hs99999901_s1, respectively.

### 2.9. Statistical Analysis

All values were presented as mean ± standard deviation. Student's *t*-test or one-way analysis of variance followed by Tukey's HSD test or Dunnett's test was performed for statistical comparisons; *p* < 0.05 was considered significant.

## 3. Results

**Figure 1:**
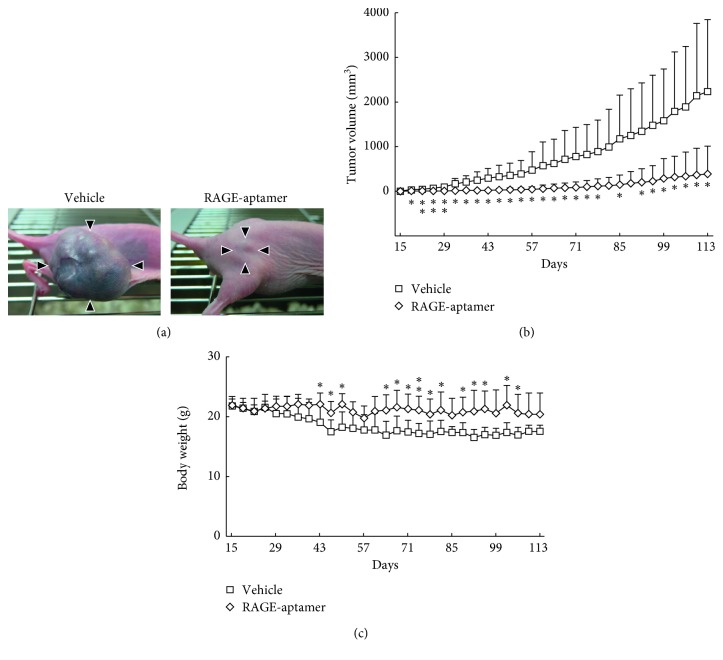
Effects of local injection of RAGE-aptamer on G361 tumor growth (a, b) in, and body weight (c) of, nude mice. Two million G361 malignant cells were intradermally administered into the upper flank of 6-week-old female athymic nude mice. Mice received local injection of RAGE-aptamer or vehicle adjacent to the tumor. Tumor size and body weight were measured twice a week till the end of experiments. Panel A shows typical photographs of G361 tumors at 113 days after tumor inoculation. Arrowheads indicate tumors. ^*∗*^*p* < 0.05 and ^*∗∗*^*p* < 0.01 compared with the values of vehicle-treated mice.

We first studied the effects of RAGE-aptamer on G361 tumor growth in nude mice. As shown in Figures 1(a) and 1(b), local injection of RAGE-aptamer adjacent to the tumor decreased the growth of G361 melanoma cells in nude mice during the experimental periods; tumor size of G361 melanoma cells reached 2,200 mm^3^ at 113 days after tumor inoculation, which was significantly reduced to ca. 390 mm^3^ by the treatment with RAGE-aptamer. On the other hand, body weight of RAGE-aptamer-treated mice was heavier than that of vehicle-treated mice. Body weight loss in tumor-bearing mice was almost prevented by RAGE-aptamer (Figure 1(c)). One mouse in vehicle-treated group died at 67 days after tumor inoculation, but the others did not. Tumor of one mouse in RAGE-aptamer-treated group was barely detectable during the experimental periods.

**Figure 2:**
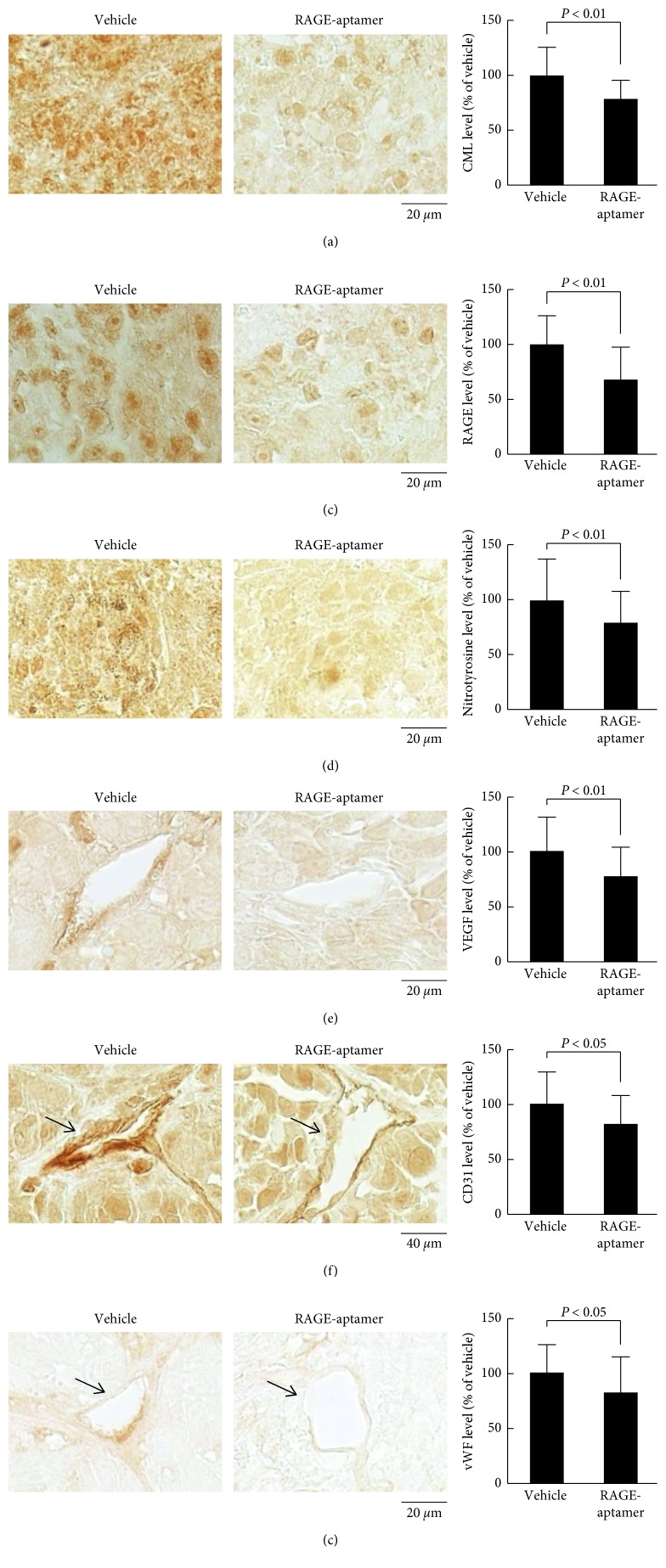
Effects of local injection of RAGE-aptamer adjacent to the tumor on CML (a), RAGE (b), nitrotyrosine (c), VEGF (d), CD31 (e), and vWF (f) expression in G361 tumors. (a–f) Each left panel shows representative photographs of immunostaining. Quantitative data are shown in each right panel. Different fields were obtained at random from each specimen, and immunoreactivity was measured by ImageJ.

Since CML has been reported to evoke numerous biological actions in a variety of organs through the interaction with RAGE via ROS generation [18, 19], we next examined the effects of RAGE-aptamer on CML-RAGE-oxidative stress axis in the G361 tumors of nude mice. As shown in Figures 2(a)–2(c), immunohistochemical analyses revealed that tumor expression levels of CML, RAGE, and an oxidative stress marker, nitrotyrosine, were significantly decreased by the long-term treatment of RAGE-aptamer. Furthermore, RAGE-aptamer treatment also reduced the expression levels of VEGF as well as markers of endothelial cells, CD31, and vWF in G361 tumors of nude mice (Figures 2(d)–2(f)).

**Figure 3:**
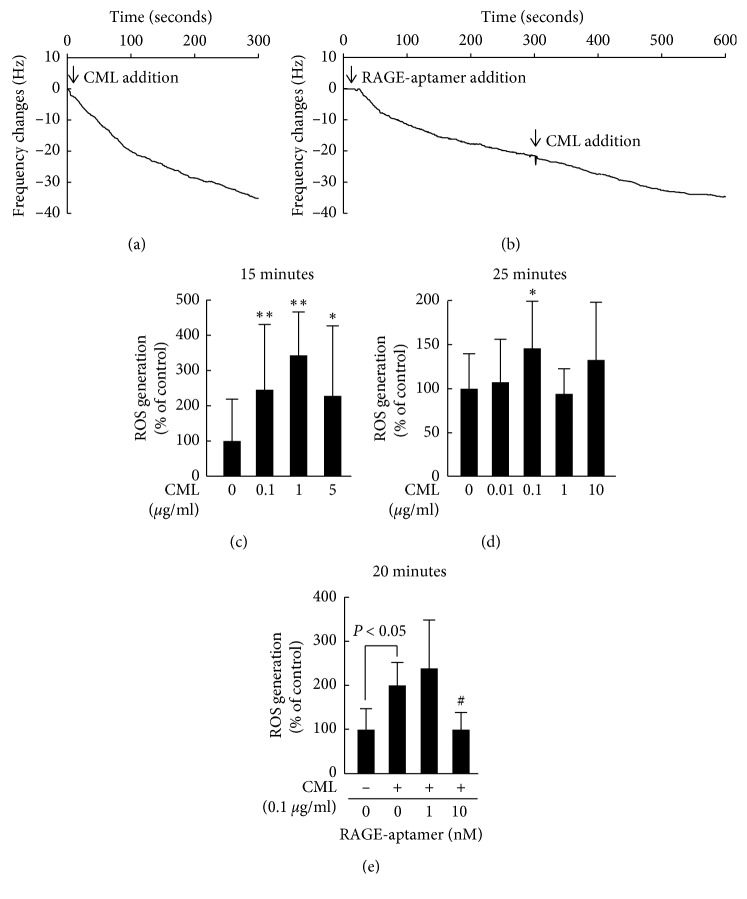
Effects of RAGE-aptamer on CML-RAGE interaction *in vitro* (a and b) and ROS generation (c–e) in CML-exposed G361 melanoma cells. a and b: representative curves of QCM sensors. Binding affinity of CML (a) or RAGE-aptamer (b) to vRAGE immobilized on a QCM surface in the presence or absence of RAGE-aptamer. *N* = 3 for each group. (c–e) G361 cells were treated with or without 10 *μ*M carboxy-H_2_DFFDA for 30∼60 minutes. Then, G361 cells were incubated with the indicated concentrations of CML in the presence or absence of the indicated concentrations of RAGE-aptamer for 25 minutes. Intracellular ROS production in G361 cells was measured with a fluorescent probe, carboxy-H_2_DFFDA. (c) *N* = 6 for each group. (d, e) *N* = 4 for each group. ^*∗*^*p* < 0.05 and ^*∗∗*^^*∗∗*^*p* < 0.01 compared with the control values without CML. ##, *p* < 0.01 compared with the values with CML alone.

We further investigated the effects of RAGE-aptamer in CML-exposed G361 melanoma cells. For this, we first examined the effects of RAGE-aptamer on CML-RAGE interaction *in vitro*. As shown in Figures 3(a) and 3(b), a sensitive 27-MHz QCM revealed that CML and RAGE-aptamer bound to vRAGE with dissociation constants (Kds) of 28.3 ± 15.8 nM and 4.44 ± 0.56 nM, respectively. However, in the presence of RAGE-aptamer, the affinity of CML to vRAGE was decreased and its Kd value was 77.1 ± 14.2 nM (Figure 3(b)).

As shown in Figure 3(c), 15 min incubation of CML increased the ROS generation in cultured G361 cells in a bell-shaped manner. Therefore, we next investigated the effect of relatively higher concentration and/or longer incubation time of CML on G361 melanoma cells. ROS production began to increase at 15 minutes after 0.1, 1, and 5 *μ*g/ml CML exposure, but returned to the basal levels by 25 minutes except for the case with 0.1 *μ*g/ml CML (Figures 3(c) and 3(d)). RAGE-aptamer dose-dependently inhibited the CML-induced increase in ROS generation in G361 cells; 10 nM RAGE-aptamer completely prevented the increase in ROS production in 0.1 *μ*g/ml CML-exposed G361 cells (Figure 3(e)).

**Figure 4:**
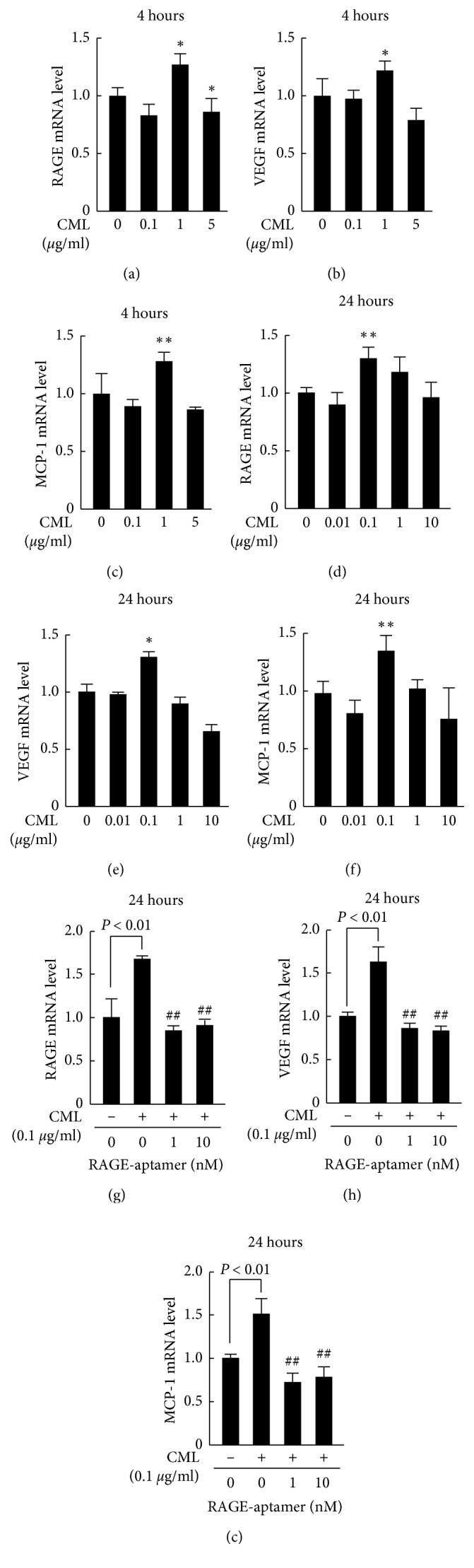
Effects of RAGE-aptamer or RAGE, VEGF, and MCP-1 mRNA levels in CML-exposed G361 melanoma cells. G361 cells were incubated with the indicated concentrations of CML in the presence or absence of the indicated concentrations of RAGE-aptamer for 4 hours (a–c) or 24 hours (d–i). Total RNAs were transcribed and amplified by real-time PCR. Data were normalized by the intensity of *β*-actin-derived signals (a–c) or 18S rRNA-derived signals (d–i) and then related to the values obtained without CML. (a, g–i) *N* = 3 for each group. (b, c) *N* = 6 for each group. (d–f) *N* = 12 for each group. ^*∗*^*p* < 0.05 and ^*∗∗*^*p* < 0.01 compared with the control values without CML. ##, *p* < 0.01 compared with the values with CML alone.

CML was also found to increase RAGE, VEGF, and MCP-1 gene expression in G361 cells in a bell-shaped manner; mRNAs reached peak levels at 4 and 24 hours after treatment with 1 and 0.1 *μ*g/ml CML, respectively (Figures 4(a)–4(f)). RAGE-aptamer at 1 or 10 nM also completely inhibited the upregulation of RAGE, VEGF, and MCP-1 mRNA levels in 0.1 *μ*g/ml CML-exposed G361 cells (Figures 4(g)–4(i)).

## 4. Discussion

We found in this study for the first time that RAGE-aptamer inhibited the binding of CML, one of the structurally identified AGEs, to vRAGE *in vitro* and local injection of RAGE-aptamer adjacent to the tumor for more than 100 days dramatically suppressed the growth of G361 melanoma tumor in nude mice during the experimental periods. At 113 days after tumor inoculation, tumor size in RAGE-aptamer-treated mice was about one-sixth of that in vehicle-treated mice. Furthermore, we found that expression levels of CML, RAGE, and nitrotyrosine, a marker of oxidative stress in the tumors, were significantly reduced by the treatment of local injection of RAGE-aptamer. Aortic and renal CML accumulation is decreased in RAGE-deficient diabetic mice, which is associated with the reduction of oxidative stress [20, 21]. Moreover, continuous intraperitoneal infusion of AGE-aptamer or RAGE-aptamer has been found to reduce tumor AGEs, RAGE, and oxidative stress levels in nude mice via blockade of the AGE-RAGE axis [17, 22, 23]. Since CML stimulates RAGE expression in both cell culture and animal models [5, 24, 25], local injection of RAGE-aptamer may decrease CML, RAGE, and nitrotyrosine expression levels in the G361 tumors by breaking the positive feedback loop among the CML-RAGE-oxidative stress system. In the present study, we found that RAGE-aptamer inhibited the CML-induced ROS generation and RAGE gene expression in cultured G361 melanoma cells, thus supporting our speculation.

CML is detected in various types of human tumors [26, 27]. CML not only induces proliferation of pancreatic ductal adenocarcinoma *in vitro* through the interaction with RAGE, but also stimulates the progression of pancreatic intraepithelial neoplasia into invasive pancreatic cancer in mice [28]. In addition, higher plasma CML levels are independently associated with increased risk of incident prostate cancer [29], whereas dietary consumption of CML is correlated with higher risk of pancreatic cancer in men as well [30]. Since we have previously shown that intraperitoneal administration of neutralizing anti-RAGE Abs inhibits the growth of G361 tumors in nude mice [31], blockade of the CML-RAGE axis could be a novel therapeutic target for malignant melanoma. Compared with neutralizing Abs, aptamers are less immunogenic, more stable with a longer shelf-life, and can be selected via high-throughput SELEX [32, 33]. Therefore, our present findings suggest that local injection of RAGE-aptamer adjacent to the tumor may be a feasible therapeutic tool for the treatment of malignant melanoma.

In this study, RAGE-aptamer injection significantly suppressed tumor-associated angiogenesis evaluated by CD31 and vWF expression in association with the reduction of VEGF levels in the G361 tumors. In addition, RAGE-aptamer inhibited the CML-induced upregulation of VEGF and MCP-1 mRNA levels in G361 melanoma cells. We, along with others, have previously shown that engagement of RAGE with CML or AGEs stimulate VEGF and MCP-1 gene expression through the interaction with RAGE via ROS generation [20, 34–37]. Tumor-associated angiogenesis plays a crucial role in malignant melanoma growth and metastasis, which is mainly regulated by VEGF and MCP-1 expression [38–40]. These observations suggest that local injection of RAGE-aptamer could inhibit tumor-associated angiogenesis by suppressing the CML-RAGE-oxidative stress axis via reduction of VEGF and MCP-1 expression in the G361 tumors of nude mice.

## 5. Limitations

We acknowledge several limitations of this study. First, in the present study, we did not use scrambled DNA-aptamer as a control agent. However, we have previously shown that (1) RAGE-aptamer significantly inhibits the development and progression of experimental diabetic nephropathy compared with control-aptamer and (2) RAGE-aptamer alone does not affect ROS generation, RAGE, VEGF, or MCP-1 gene expression in G361 melanoma cells or human mesangial cells [16, 17]. Furthermore, in this study, local injection of RAGE-aptamer significantly inhibited the decrease in body weight in tumor-bearing mice. Therefore, it is unlikely that aptamer itself had nonspecific and toxic effects on G361 melanoma in nude mice. Second, although the effects of local injection of RAGE-aptamer on tumor growth were drastic, we could not clarify here whether RAGE-aptamer treatment could prolong the survival of G361 tumor-bearing mice because only one mouse in the vehicle-treatment group died during the experimental periods over 113 days. Third, it is interesting to further examine the role of other RAGE ligands, such as high mobility group box-1 and S100 proteins [1] in melanoma growth in nude mice. Fourth, since long-term local injection of RAGE-aptamer dramatically attenuated the growth of G361 melanoma cells, we could not obtain enough amount of proteins to perform western blot analyses from the tumors. Therefore, the results of immunohistochemical staining could not be confirmed by western blot analyses. Fifth, in this study, effects of CML on RAGE, VEGF, and MCP-1 mRNA levels were not dose dependent. Relatively higher concentrations of CML may have toxic effects on cultured G361 melanoma cells. This is one possible reason why CML increased the RAGE, VEGF, and MCP-1 mRNA levels in a bell-shaped manner. Sixth, we have previously shown that RAGE-aptamer not only inhibits VEGF and MCP-1 gene expression in both AGE-exposed G361 melanoma cells and endothelial cells, but also suppresses THP-1 cell adhesion to, and tube formation of, AGE-exposed endothelial cells, thus suggesting the pathological role of VEGF and MCP-1 protein expression in AGE-RAGE-induced neoangiogenesis and macrophage infiltration in G361 melanoma [17]. Therefore, it would be helpful to examine the effects of RAGE-aptamer on RAGE, VEGF, and MCP-1 proteins levels by western blot analyses. Seventh, we have previously found that DNA-aptamer raised against AGEs significantly attenuates the growth of G361 melanoma in female nude mice [22]. This is a reason why we chose female but not male athymic mice in the present animal experiments. Eighth, although we did not show the data of negative control in QCM experiments, we, along with others, have already shown that RAGE is a cell surface receptor for AGEs, including CML, but not for nonmodified BSA [16–18].

## 6. Conclusions

Our present findings suggest that long-term local injection of RAGE-aptamer adjacent to the tumor could inhibit melanoma growth in nude mice partly by suppressing tumor angiogenesis via blockade of the CML-RAGE interaction. Local injection of RAGE-aptamer may be a feasible therapeutic tool for the treatment of malignant melanoma.

## Data Availability

The data used to support the present findings are included in this published article.
